# Mood Disorders in the Wake of Traumatic Brain Injury: A Systematic Review

**DOI:** 10.7759/cureus.62524

**Published:** 2024-06-17

**Authors:** Yaneisi Palou Martinez, Divine Besong Arrey Agbor, Priyanka Panday, Samrah Ejaz, Simhachalam Gurugubelli, Suviksh K Prathi, Tuheen Sankar Nath

**Affiliations:** 1 Research and Medicine, California Institute of Behavioral Neurosciences & Psychology, Fairfield, USA; 2 Clinical Research and Internal Medicine, California Institute of Behavioral Neurosciences & Psychology, Fairfield, USA; 3 Internal Medicine, Richmond University Medical Center, Staten Island, USA; 4 Research, California Institute of Behavioral Neurosciences & Psychology, Fairfield, USA; 5 Internal Medicine, California Institute of Behavioral Neurosciences & Psychology, Fairfield, USA; 6 Internal Medicine, Memorial Healthcare, Gulfport, USA; 7 Research, St. George's University School of Medicine, St. George's, GRD; 8 Surgical Oncology, Tata Medical Centre, Kolkata, IND

**Keywords:** anxiety, post-traumatic stress disorder, major depression, mood disorders, traumatic brain injury

## Abstract

Traumatic brain injury (TBI) frequently leads to a myriad of long-term consequences, among which mood disorders present a significant challenge. This systematic review delves into the complex interplay between TBI and subsequent mood disorders, focusing on research studies conducted over the past decade. Encompassing an age range from 12 years old to older adults (60+ years), our review aims to elucidate the epidemiological patterns, neurobiological mechanisms, and psychosocial factors that contribute to the development of mood disorders following TBI. By synthesizing the current literature, we seek to uncover the prevalence and clinical implications of this often-under-recognized comorbidity. For the quality appraisal of the reviewed articles, the Newcastle-Ottawa risk-of-bias tool and Scale for the Assessment of Narrative Review Articles (SANRA) checklist were employed. Ultimately, this review endeavors to provide a comprehensive understanding of the intricate relationship between TBI and mood disorders, offering insights crucial for improved management and intervention strategies in affected individuals.

## Introduction and background

Traumatic brain injury (TBI) is now being characterized as a long-term health condition, as research suggests that the health consequences of TBI can endure over an extended period, especially among individuals who have undergone moderate to severe TBI [1,2]. Beyond the immediate physical and cognitive challenges, TBI often casts a long shadow on an individual's emotional well-being. Mood disorders, including depression, anxiety, post-traumatic stress disorder (PTSD), substance abuse, and psychotic disorders, frequently emerge as silent companions to the physical aftermath of TBI, complicating the path to recovery and rehabilitation [3,4]. While most of the research concerning TBI outcomes and its long-term consequences primarily centers on moderate-to-severe cases, it is worth noting that mild TBI (mTBI) accounts for the highest frequency of visits to the emergency department (ED) [5].

Even though there are signs pointing to a heightened likelihood of psychiatric disorders in individuals with TBI, the connection between mTBI and the increased susceptibility to concurrent psychiatric conditions remains inadequately investigated and understood [4,6]. Furthermore, limited knowledge exists regarding how depression affects health-related outcomes and the pace of recovery following an mTBI [7]. Per recent studies, pre-existing psychiatric conditions worsen the severity of both TBI symptoms and psychiatric symptoms three to six months following the injury. This underscores the significance of conducting a thorough medical history assessment during diagnosis to gain better insights into the psychiatric outcomes associated with TBI [6].

In this systematic review, we will examine the existing high-quality body of literature to provide a comprehensive synthesis of the current knowledge on the topic. We will explore the epidemiological patterns, neurobiological underpinnings, and psychosocial factors contributing to mood disorders in TBI patients, aiming to unravel the intricate relationship between TBI and mood disorders and shed light on the prevalence and clinical implications of this often-under-recognized comorbidity. Understanding the synergy between TBI and mood disorders is paramount not only for healthcare providers but also for policymakers, researchers, and the individuals and families affected. It can inform tailored interventions, improve the quality of life for TBI survivors, and reduce the societal burden of mood disorders in this population. It is our hope that this endeavor will contribute to a deeper understanding of the complex interplay between TBI and mood disorders, ultimately improving the lives of those affected by these challenging conditions.

## Review

Methods

The guidelines and principles outlined in the Preferred Reporting Items for Systematic Reviews and Meta-Analyses (PRISMA) were followed during the evolution of this investigation [8].

Inclusion Criteria

The inclusion criteria for our systematic review comprise clinical trials, meta-analysis, randomized controlled trials, reviews, and systematic reviews conducted within the past decade investigating mood disorders subsequent to TBI across a diverse age range, spanning from adolescence to older adulthood, and published in English only. Included studies must specifically address mood disorders resulting from TBI, encompassing a spectrum of severity from mild to severe TBI.

Search Strategy and Selection of Articles

Our search strategy involved querying the PubMed and Google Scholar databases utilizing keywords and Medical Subject Heading (MeSH) to identify relevant studies published within the past decade (2013-2023). Subsequently, the retrieved articles were exported to EndNote and finally compiled in Microsoft Excel (Microsoft Corporation, USA) for systematic organization, with duplicates promptly eliminated. Studies deemed irrelevant were screened based on their title and abstract and excluded from consideration. A summary of our PubMed search is presented in Table 1 for reference.

**Table 1:** PubMed search strategy MeSH: Medical Subject Heading, Majr: major topic

PubMed search	Major topic MeSH terms	Filters	Results
Concept 1	( "Brain Injuries, Traumatic/complications"[Majr] OR "Brain Injuries, Traumatic/diagnosis"[Majr] OR "Brain Injuries, Traumatic/diagnostic imaging"[Majr] OR "Brain Injuries, Traumatic/epidemiology"[Majr] OR "Brain Injuries, Traumatic/etiology"[Majr] OR "Brain Injuries, Traumatic/physiopathology"[Majr] OR "Brain Injuries, Traumatic/psychology"[Majr] OR "Brain Injuries, Traumatic/rehabilitation"[Majr] OR "Brain Injuries, Traumatic/therapy"[Majr] )	Free full text, clinical trial, meta-analysis, randomized controlled trial, review, systematic review, English, from 2013 to 2023	1266
Concept 2	( "Mood Disorders/diagnosis"[Majr] OR "Mood Disorders/epidemiology"[Majr] OR "Mood Disorders/ethnology"[Majr] OR "Mood Disorders/etiology"[Majr] OR "Mood Disorders/prevention and control"[Majr] OR "Mood Disorders/psychology"[Majr] OR "Mood Disorders/rehabilitation"[Majr] OR "Mood Disorders/therapy"[Majr] )	Free full text, clinical trial, meta-analysis, randomized controlled trial, review, systematic review, English, from 2013 to 2023	5746
Combined search	Concept 1 AND Concept 2	Free full text, clinical trial, meta-analysis, randomized controlled trial, review, systematic review, English, from 2013 to 2023	96

Results

**Figure 1:**
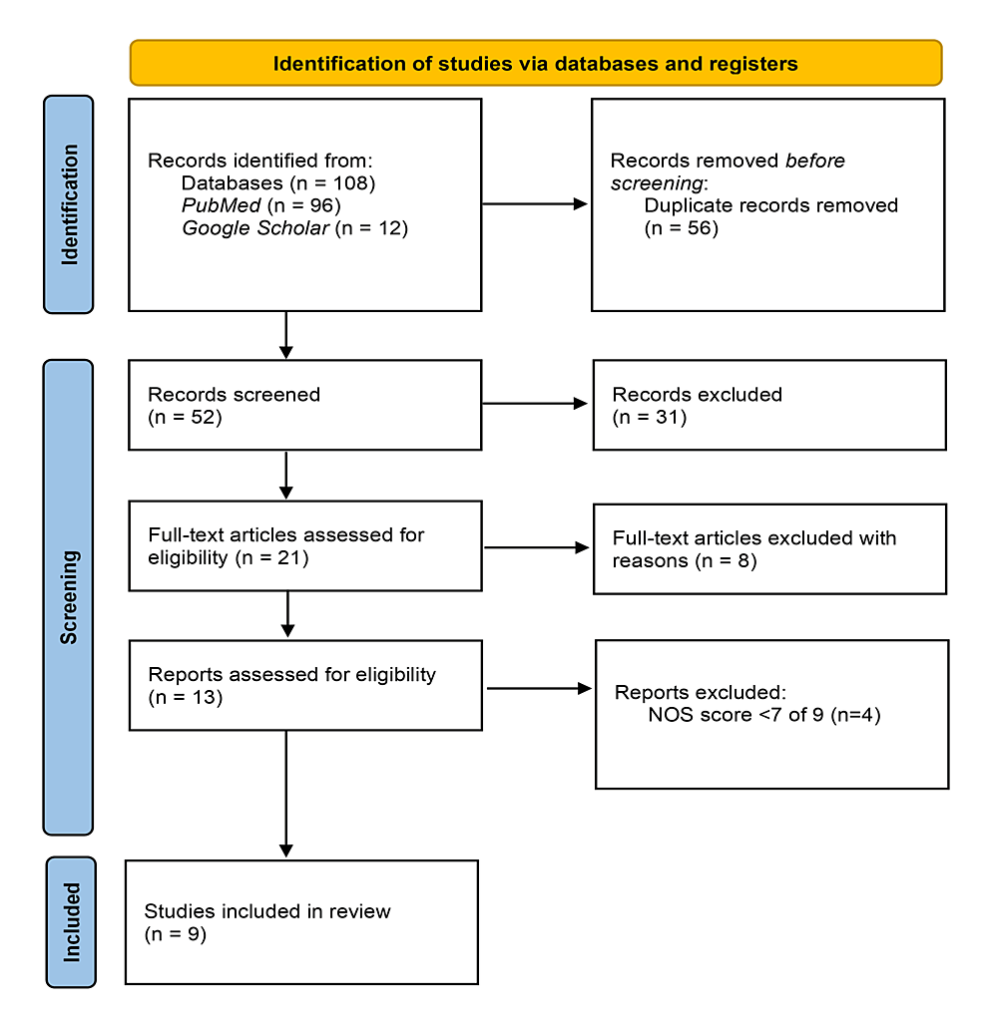
Flowchart of article selection Adapted from PRISMA 2020 [8]. PRISMA: Preferred Reporting Items for Systematic Reviews and Meta-Analyses

The search strategy identified 108 articles from PubMed and Google Scholar databases, using relevant keywords and Medical Subject Heading (MeSH). After removing 56 duplicates, 52 records remained for screening. From these, 39 articles were excluded based on titles, abstracts, and detail-inclusive criteria. The full texts of 13 articles were assessed for eligibility, resulting in the exclusion of four articles after quality appraisal. Ultimately, nine studies were included in the final review. Figure 1 shows the Preferred Reporting Items for Systematic reviews and Meta-Analyses (PRISMA) flowchart of the studies included for reference.

Quality Assessment of Individual Studies

Upon the quality assessment of individual articles, only nine studies met the criteria for inclusion in our review. Tables 2-3 display in detail the chosen studies.

**Table 2:** Summary of the Newcastle-Ottawa risk-of-bias tool for observational studies

Selection	Spitz et al., 2017 [9]	Thomas et al., 2021 [10]	Bombardier et al., 2016 [11]	Jolly et al., 2019 [12]	Stein et al., 2019 [13]	Shi et al., 2023 [14]
Representativeness of the intervention cohort	1	1	1	1	1	1
Selection of the non-intervention cohort	1	1	1	1	1	1
Ascertainment of intervention	1	1	1	1	1	1
Demonstration that outcome of interest was not present at start of study	0	0	0	1	0	0
Comparability						
Study controls for age	1	1	1	1	1	1
Study controls for any additional factors	1	1	1	1	1	1
Outcome						
Assessment of outcome	1	1	1	1	1	1
Was follow up long enough for outcomes to occur?	1	1	0	0	1	1
Adequacy of follow up of cohorts	0	0	1	1	0	0
Total	7/9	7/9	7/9	8/9	7/9	7/9
Quality	Medium	Medium	Medium	High	Medium	Medium

**Table 3:** Summary of the SANRA Checklist SANRA: Scale for the Assessment of Narrative Review Articles

	Ponsford et al., 2018 [15]	Deck et al., 2021 [16]	Howlett et al., 2022 [17]
Justification of article’s importance	2	2	1
Statement of concrete aims or formulation of questions	1	2	1
Description of the literature search	2	1	2
Referencing	2	2	2
Scientific reasoning	2	2	2
Appropriate presentation of data	1	1	2
Total	10	10	10

Discussion

In our systematic review centered on TBI and mood disorders, our findings shed light on a crucial facet of psychiatric outcomes subsequent to TBI. Specifically, our analysis indicates that depressive disorders emerge as the foremost prevalent psychiatric consequences following TBI, with heightened within the first year. The summary of the nine selected articles showcases the most significant aspects of our findings.

White-Matter Abnormalities in TBI

Spitz et al.'s research in 2017 was groundbreaking as it was the initial investigation to explore the connection between mood disorders and white-matter issues in patients who had experienced various degrees of TBI [9]. The study employed a standardized psychiatric interview to gather data from a group of 29 participants, who were part of a larger prospective study focused on the development of psychiatric conditions in the aftermath of complicated, mild to severe TBI, and compared to a group of 23 healthy control individuals [9]. The severity of the injury and other background factors showed no significant distinctions between TBI participants with or without a mood disorder. White matter microstructure was evaluated through diffusion tensor tractography, employing both atlas-based tract-averaged and along-tract methodologies [9]. The statistical analysis was carried out using Stata Statistical Software (College Station, TX: StataCorp LLC), specifically version 14.1 [18]. After the tract-averaged regressions for each white matter pathway, adjustments for multiple comparisons were made by maintaining a false discovery rate (FDR) control at a significance level of p < 0.05 [19]. Significant statistical disparities were observed when comparing the healthy control participants in the imaging data to the TBI groups. The two most anterior sections of the corpus callosum showed a decrease in fractional anisotropy (FA), and this reduction was strongly linked to mood disorders following TBI [9].

In the study, the superior and inferior genu, along with the rostrum of the corpus callosum, which sends fibers to the prefrontal cortex, exhibited the most robust connection with mood disorders. This aligns with the prevailing body of research in the TBI domain, which has consistently linked frontal brain abnormalities to mood disorders following injuries [20,21]. Despite employing an impartial tractography method and mitigating the risk of type I errors through FDR adjustment, the present study investigated mood within a rather limited sample of individuals who had experienced TBIs [9]. Nevertheless, it is important to exercise caution since the TBI-Mood group in the current study exhibited a higher incidence of pre-existing substance-use disorders in comparison to the TBI-No Mood participants [9]. This study would have benefited from a larger sample size. 

Regional Cerebral Blood Flow and Emotional Symptoms

Sixteen individuals who reported persistent neurological symptoms following a TBI, and 16 healthy control participants who were matched in terms of age and gender for comparison were included in Thomas et al.'s 2021 study. Regional cerebral blood flow (CBF) using a technique known as pseudo-continuous arterial spin labeling (PCASL) and brain volume were assessed through T1-weighted imaging data. Through voxel-wise regression analysis, it was discovered that reduced CBF in specific brain regions, particularly the hippocampus and the rostral anterior cingulate, was linked to increased self-reported symptoms of anger, anxiety, and depression [10]. These negative associations were compared with previous studies [22,23], which hypothesized that damage to small blood vessels resulting from TBI could potentially result in reduced blood flow to specific brain regions. This diminished perfusion may become more pronounced during the chronic phase of TBI and could be associated with the emergence of emotional symptoms [10]. We acknowledge their contribution for being the initial team to demonstrate a notable adverse correlation between CBF and symptoms assessed by the patient-reported outcome measurement information system (PROMIS) in individuals with TBI, however, there is a lack of validation in a more uniform population and the small power limited the study. 

Trajectories of Depression Following TBI

Bombardier et al.'s 2016 study revealed that the predominant trajectory within the initial year following TBI was characterized by low depression, encompassing 70.1% of the participants [11]. This discovery aligns with the rates of non-depressive outcomes observed in earlier one-year prospective prevalence studies on TBI, falling within the range of 57-71% [20,24-27]. From 2001 to 2006, 559 individuals with mild to severe TBIs were recruited from inpatient facilities at Harborview Medical Center in Seattle, Washington. These participants underwent monthly evaluations using the nine-item Patient Health Questionnaire (PHQ-9) to assess depression for the first six months, followed by assessments every two months for a year post-injury [11]. Post-traumatic stress disorder (PTSD), known for its high prevalence and significance in TBI, was analyzed separately [25]. The remaining participants were grouped under "other mental health disorders."

The study utilized multinomial logistic regression to identify trajectory classes based on demographic, psychiatric history, and clinical variables. Among the identified trajectories, the second most common one was characterized by delayed depression, representing 13.2% of the total sample and 15.9% of those with low depression scores initially. Despite starting with low PHQ-9 scores, this group showed a higher likelihood of having pre-injury mental health and substance use risk factors. The other trajectory categories were depression recovery (10.4% of participants) and persistent depression (6.3%). Individuals in the persistent depression group were more likely to be aged 30-44 and had higher rates of pre-TBI mental health and substance abuse issues compared to those in the low depression trajectory. Despite appropriate treatment for individuals persistently experiencing depression in this study, controlled trials of depression treatment in individuals with TBI indicate that antidepressants and cognitive-behavioral therapy have limited effectiveness [28,29]. In addition, it should encompass measures of other mental health conditions commonly associated with TBI, such as various mood, anxiety, and substance abuse disorders [26]. This study was conducted to address the knowledge gap pertaining to the progression of depressive symptoms following a TBI, enhancing our capacity to comprehend causal factors, forecast outcomes, and further interventions.

Dopaminergic Abnormalities in TBI

In Jolly et al.'s 2019 cross-sectional study, 12 individuals with a sole moderate-to-severe TBI underwent assessment through [11C] 4‐propyl‐9‐hydroxynaphthoxazine (PHNO) positron emission tomography (PET), structural T1 magnetic resonance imaging (MRI), diffusion tensor imaging (DTI), and neuropsychological tests. A group of 26 controls matched for age underwent imaging utilizing PET with [11C]PHNO and structural MRI. TBI patients, along with an additional set of 32 controls, also participated in DTI and neuropsychological assessments [12]. TBI patients were categorized as either having major depressive disorder (MDD) or exhibiting no depressive symptoms, as determined through the Structured Clinical Interview for Diagnostic and Statistical Manual of Mental Disorders, fourth edition (DSM-IV)-TR AXIS I Disorders, version I/NP (SCID) [30]. The statistical analysis proceeded in two steps. Initially, a comparison was made between all TBI patients and controls to discern differences associated with TBI. Subsequently, an examination was conducted to explore the connection between TBI and the presence of MDD [12].

Reduced [11C]PHNO non-displaceable binding potential (BPND) was identified in the caudate across all TBI patients in comparison to controls. TBI-MDD patients exhibited lower [11C]PHNO BPND in the caudate, while TBI-NON patients showed increased [11C]PHNO BPND in the amygdala compared to controls. No significant differences in [11C]PHNO BPND were noted between TBI-MDD and TBI-NON individuals [12]. The binding potential of [11C]PHNO in the caudate showed a correlation with fractional anisotropy within the nigro-caudate tract. In addition, DTI revealed evidence of axonal injury post-TBI, with abnormally low FA observed in the uncinate fasciculus and cingulum, particularly affecting the uncinate in TBI-MDD patients [12]. Their results build upon prior research that has shown abnormalities in the dopaminergic system following TBI [31,32]. Findings that duplicate the discovery of diminished D2 receptor binding in the caudate. However, they also offer indications of variability in the impact of TBI on dopamine receptors, as [11C]PHNO binding increased in patients without MDD in the amygdala. The relatively small sample sizes in the TBI-patient groups, potentially affecting the ability to identify differences, should be considered a limitation for this study; however, their results indicate some potentially intriguing D2/D3 regions worth further exploration in significantly larger patient cohorts.

PTSD and MDD

Stein et al. conducted a longitudinal cohort study spanning from February 2014 to May 2018 [13]. The study included 1,155 individuals with mTBIs and 230 individuals aged 17 years and older who had non-head orthopedic trauma injuries. These participants were drawn from 11 level 1 trauma centers across the United States and were assessed in the emergency department based on an ED arrival Glasgow Coma Scale (GCS) score falling within the range of 13 to 15. The mean age of the participants was 40.5 years with a standard deviation of 17.2 years, while the average years of education were 13.6 with a standard deviation of 2.9. Out of the 1,155 individuals, 752 (65.1%) were male, 881 (77.2%) identified as White, and 245 (21.4%) identified as Hispanic [13]. A psychiatric history was present in 239 patients (21%). Symptoms of PTSD and MDD were evaluated using the PTSD Checklist for DSM-5 and the Patient Health Questionnaire-9 [13].

The six-month prevalence rate of MDD among the participants was 9%, comparable to the 7% reported in a Dutch ED study but slightly lower than the 21% documented in a US study involving TBI patients treated in a single level 1 trauma center [33,34]. In line with previous research indicating that a pre-existing history of MDD heightens the likelihood of experiencing MDD following TBI, these findings demonstrated that a pre-injury mental health issue was an especially robust risk factor for developing post-injury PTSD or MDD [34]. The risk factors (e.g., age, sex, race, and education) for a likely MDD were comparable to PTSD, except for the fact that the cause of injury did not show an association with an elevated risk. Additional factors related to the injury, such as the duration of loss of consciousness, post-traumatic amnesia, the presence of brain injury on a computed tomography (CT) scan, or hospitalization (with or without intensive care unit admission), did not demonstrate an association with the risk of PTSD or MDD [13].

Despite the potential constraint of this study lying in its concentration on just two (PTSD and MDD) out of numerous potentially significant mental disorders, this study is fortified by its meticulous multisite sampling methodology, the ongoing assessment over time, and the substantial magnitude of its participant cohort, collectively enhancing the robustness and reliability of its findings.

Understanding Psychiatric Disorders Post-TBI

Ponsford et al.'s 2018 article elucidated the occurrence and progression of psychiatric disorders following TBI [15]. The prevalence of psychiatric disorders following a TBI shows significant variability, ranging from 18.3% to 83.3% in the months and years following the injury [15,35], which is affected by factors such as the recruitment methods in studies, diagnostic approaches, the severity of the injury, and the duration of time since the injury occurred. Partially considering the demographic distinctions between the TBI population, these individuals are generally at a higher likelihood of having a preexisting alcohol use disorder [26]. This could indicate a connection between alcohol, engaging in risky behaviors, and the occurrence of injuries. The prevalence of anxiety and mood disorders prior to the injury was 21.7% and 23.0%, respectively. Research that retrospectively explores post-injury psychiatric disorders has indicated rates varying from 48.3% to 83.3%, suggesting that these disorders might persist beyond the initial year following the injury [36,37].

After experiencing a TBI, mood disorders emerge as the most prevalent psychiatric condition, surpassing the rates observed in the general population [26,38], and often arise in the absence of a preinjury psychiatric background and co-occur with anxiety disorders in about 75% of cases [20,39]. The most prevalent diagnosis is MDD. Research predominantly centered on mild TBI cases indicates that MDD typically emerges within three months post-injury [27]. However, a recent study concentrating on moderate to severe injuries found that symptom onset is often delayed, frequently occurring between six and 12 months after the injury [26]. There is an indication that mTBI could potentially lead to the development of acute stress disorder and PTSD, especially in cases where the loss of consciousness and post-traumatic amnesia are brief, allowing for partial or complete encoding of the traumatic event [40]. The incidence of acute stress disorder resulting from a minor TBI has fluctuated between 4.6% and 21.2%. There is some indication that acute stress disorder often transitions into PTSD following mTBI [41]. The initiation of PTSD reached its highest point between six and 12 months after the injury. A majority of PTSD cases (66.7%) experienced a delayed onset [15].

It was concluded that substance use, although prevalent prior to the injury, decreases post-injury. Current studies indicate that the occurrence of psychotic, eating, somatoform, and adjustment disorders among individuals with TBI does not surpass the rates observed in the general population [15]. They explore a diverse range of psychiatric conditions beyond anxiety and mood disorders, including substance use, psychotic, eating, and somatoform disorders, as well as adjustment disorders, throughout the course following a TBI, enhancing the comprehensiveness of their study. 

Depression Incidence Post-Cerebral Concussions in Athletes

Deck et al. published a comprehensive review of empirical findings derived from diverse studies published on an annual basis from 2013 to 2019, encompassing athletes and professional athletes, with a focus on youth, adolescents, and retired players [16]. The primary objective is to ascertain whether the occurrence of depression is elevated subsequent to cerebral concussions. These studies exhibit a notable risk of bias due to their dependence on self-reported concussions, variations in the definitions of depression, and potential unmeasured confounding factors. As a result, the precise connection, between concussion and depression remains uncertain [16].

A prospective cohort investigation conducted in 2019 among high school students included a total of 3427 participants from 98 schools, who engaged in physical activity for a minimum of 60 minutes on five or more days per week or were members of at least one sports team [16]. In contrast to the overall cohort of enrolled students, 664 individuals who self-reported experiencing a concussion in the preceding 12 months exhibited an increased adjusted odds ratio (aOR) for depressive symptoms (aOR = 1.5; 95% confidence interval [CI], 1.1-1.9) [42].

In a prospective cohort study conducted in 2016, the influence of concussive incidents on the occurrence of depression was examined among active semiprofessional and professional football players with a history of ≥1 concussion. The study involved 27 participants who responded to an anonymous online survey, incorporating the revised version of the Center for Epidemiologic Studies Depression Scale (CESD-R) to assess the degree of depression, with clinical depression defined as a score of ≥16. Athletes who experienced three or more concussions recorded notably higher scores on the CESD-R compared to those with two or fewer concussions (average score, 24 vs. 15.6; P = .03) [43].

In a 2017 case-control study exploring the long-term health outcomes of 52 retired Scottish male rugby players with a history of mild concussion compared to age-matched males without a history of concussion (N = 29), no statistically significant difference was observed in the mean Hospital Anxiety and Depression Scale scores between the two groups (2.8 ± 2.1 vs. 2.6 ± 2.8; P = .941) [44]. 

TBI and Psychiatric/Neurobehavioral Problems

Howlett et al. provided an overview of the relationship between TBI and psychiatric/neurobehavioral problems, emphasizing the shift in focus from mortality rates to the cognitive, affective, and behavioral consequences of TBI across all severity levels [17]. It discusses how moderate and severe TBIs can lead to personality changes, while mild TBIs are associated with affective symptoms, suicidality, and the exacerbation of psychiatric disorders like PTSD and MDD [17]. The epidemiology and classification of TBI are outlined, noting the significant number of cases and the challenges in diagnosing and categorizing TBIs due to their varying severity and context (military, civilian, and athletic). The article also explores persistent post-concussive symptoms, which can include somatic, cognitive, and emotional/behavioral issues, and highlights the complexities of understanding their etiology.

Publicized case reports have brought to light worries that repeated head injuries could lead to chronic traumatic encephalopathy (CTE), a neurodegenerative disease [45]. CTE is believed to result in a variety of cognitive, psychiatric, and behavioral issues, collectively known as traumatic encephalopathy syndrome (TES) [45,46]. The discussion emphasizes the need for longitudinal studies to confirm the relationship between repetitive head impacts and neurodegenerative disorders. In addition, it addresses affective symptoms and disorders post-TBI, noting their prevalence across all severity levels and the impact on disability and quality of life [47,48]. The article further delves into the association between TBI and PTSD, highlighting the increased risk of PTSD following TBI, particularly in military populations, and the potential mechanisms underlying this relationship. TBI has the potential to harm neural pathways responsible for controlling fear responses [49]. In addition, cognitive decline following TBI might diminish individuals' ability to cope and utilize effective cognitive strategies [50]. Furthermore, inflammatory reactions subsequent to TBI may exacerbate mental health challenges [51]. Suicidality is also explored as a significant sequela of TBI, with several studies demonstrating associations between TBI and suicidal ideation/behaviors, both in military and civilian populations.

Finally, the article briefly discusses other manifestations of TBI, including personality changes, externalizing psychopathology like substance misuse, and their impact on post-TBI outcomes. It emphasizes the need for further research to better understand and address the complex interplay between TBI and mental health consequences across various contexts [17]. Cognitive impairments, although a crucial aspect of TBI, fall beyond the purview of this review. 

Post-mTBI Symptomatology in Veterans

Shi et al. published a retrospective cross-sectional analysis that examined the symptomatology of MDD and PTSD following mild TBI in veterans [14]. It aims to identify central symptoms and compare networks of participants with positive and negative mTBI screens. The study also explores the associations between these symptoms and clinical covariates (anxiety, insomnia, resilience, and emotional support). The findings highlight the importance of understanding these mental health conditions in the context of mTBI to enhance screening, monitoring, and treatment strategies. This research involved 2,797 veterans who received treatment at the Veterans Affairs (VA) San Diego Healthcare System (VASDHS) from July 1, 2014, to November 22, 2017. Most of the individuals involved were men (84.7%) with an average age of 36.3 years (standard deviation = 9.0) [14]. Descriptive statistics characterized the study population, while chi-square tests were used to compare PTSD and MDD prevalence rates and their co-occurrence between samples with and without mTBI [14].

The prevalence of mTBI in the general adult population is approximately 0.6%, but among military veterans, who are frequently exposed to combat situations, the rates are substantially higher, ranging from 12% to 23% [52-55]. Experiencing a sense of detachment/isolation and struggling with focus emerged as central nodes, aligning with conclusions drawn from earlier research, and indicating their potential as screening and treatment targets for post-mTBI PTSD and MDD [56-58]. In addition, overlapping symptoms such as sleep and concentration problems played a significant role in comorbidity. Interestingly, they found no significant differences in network structure between veterans with and without mTBI, suggesting that while mTBI increases the likelihood of severe PTSD and/or MDD, it does not substantially modify the structure of symptoms [14]. Despite the strengths of the study, including its quantitative evidence and large sample size, there are limitations, such as the cross-sectional nature of the data and potential missing influential factors in the network. Future research should address these limitations by focusing on longitudinal data and incorporating more potential influential factors. In addition, examining the network structure in a larger veteran sample would further enhance understanding in this area [14].

Limitations

It is essential to acknowledge the limitations of our review. Our focus solely on mood disorders may have overlooked valuable insights into the broader behavioral and cognitive consequences of TBI. Furthermore, our restriction to free, English-language articles published within the past decade limited the scope of our analysis, resulting in a relatively small pool of studies. Despite these limitations, our systematic review provides crucial insights into the intricate interplay between TBI and mood disorders, offering valuable directions for future research and clinical interventions in this complex area of study.

## Conclusions

Our systematic review underscores the significant consequences of TBI on mental health, with a particular emphasis on mood disorders. Our findings indicate that mood disturbances emerge as the most prevalent psychiatric outcome post-TBI, often occurring independently of preinjury psychiatric history and frequently co-occurring with anxiety disorders. Notably, our analysis reveals a distinct trajectory characterized by a heightened prevalence of depression within the initial year following TBI. Furthermore, we elucidate the neurobiological mechanisms underpinning mood disorders post-TBI, identifying frontal brain abnormalities and dysregulation in the dopaminergic system as key contributing factors. In addition, our review highlights the increased risk of PTSD following TBI, especially among military populations, underscoring the unique challenges faced by this demographic. Future research endeavors should aim to integrate data obtained from a more substantial cohort of individuals with traumatic brain injury in comparison to the broader population, or alternatively, consider conducting meta-analyses to enhance statistical power and robustness.
